# Airway Management in Adult Intensive Care Units: A Survey of Two Regions in China

**DOI:** 10.1155/2022/4653494

**Published:** 2022-11-21

**Authors:** Sheng Zhang, Jintuan Lin, Xiaoyan Diao, Wenjian Shi, Lei Huang

**Affiliations:** ^1^Department of Critical Care Medicine, Peking University Shenzhen Hospital, Shenzhen, Guangdong, China; ^2^Department of Critical Care Medicine, Shenzhen Sixth People's Hospital, Shenzhen, Guangdong, China; ^3^Obstetrics Department, Maternal and Child Health Hospital of Xinjiang Uygur Autonomous Region, Urumchi, Xinjiang, China; ^4^Department of Anesthesiology, People's Hospital of Xinjiang Uygur Autonomous Region, Urumchi, Xinjiang, China

## Abstract

The critical medicine residency training in China started in 2020, but no investigation on the practice of tracheal intubation in ICUs in China has been conducted. A survey was sent to the adult ICUs in public hospitals in Shenzhen (SZ) city and Xinjiang (XJ) province using a WeChat miniprogram to be completed by intensive care physicians. It included questions on training on intubation, intubation procedures, and changes in the use of personal protective equipment due to COVID-19. We analyzed 301 valid questionnaires which were from 72 hospitals. A total of 37% of respondents had completed training in RSI (SZ, 40% vs. XJ, 30%; *p* = 0.066), and 50% had participated in a course on the emergency front of the neck airway (SZ, 47% vs. XJ, 54%; *p* = 0.256). Video laryngoscopy was preferred by 75% of respondents. Manual ventilation (56%) and noninvasive positive pressure ventilation (34%) were the first-line options for preoxygenation. For patients with a high risk of aspiration, nasogastric decompression (47%) and cricoid pressure (37%) were administered. Propofol (82%) and midazolam (70%) were the most commonly used induction agents. Only 19% of respondents routinely used neuromuscular blocking agents. For patients with difficult airways, a flexible endoscope was the most commonly used device by 76% of respondents. Most participants (77%) believed that the COVID-19 pandemic had significantly increased their awareness of the need for personal protective equipment during tracheal intubation. Our survey demonstrated that the ICU doctors in these areas lack adequate training in airway management.

## 1. Introduction

Acute airway management is often the responsibility of critical care and emergency doctors in China. Hence, endotracheal intubation (EI) is a core skill for intensive physicians, which requires practical training and teamwork.

EI for critical patients always is a routine and dangerous performance in an emergency room and intensive care unit (ICU). Critically ill patients are physiologically very different from patients who undergo intubation in an operating room (OR) for various reasons, such as the presence of preexisting hypoxemia, fragile hemodynamic state, metabolic acidosis, and high intracranial pressure. New data showed that the incidence of adverse events associated with airway management in ICUs was higher by a factor of 55 when compared with that in ORs [1]. Complications associated with intubation have been reported to occur in up to 45% of critically ill patients, with cardiovascular instability occurring in 42%, severe hypoxemia in 9.3%, aspiration in 3.9%, cardiac arrest in 3.1%, and cardiac arrhythmia in 5.6% [2]. For critically ill patients, the main risk of peri-intubation adverse events arises from the failure of the first attempt at EI, in addition to “physiologically difficult airway.” An adaptive procedure should include preintubation patient assessment, preoxygenation, rapid intubation procedures, and other recommended measures to improve the intubation success rate and minimize intubation-related issues. However, there are some controversies regarding the patient's position, cricoid pressure, and the use of neuromuscular blocking agents (NMBAs) [3, 4].

Nevertheless, the procedures and complication rates for managing the emergency airway in the ICU may vary from one region to another. In particular, residency training of critical care medicine in China was just established in 2020, and intensivists have different professional backgrounds; moreover, there is no uniform training program for airway management in China. Shenzhen (SZ) is one of the most economically developed cities in China and has relatively adequate medical resources. Xinjiang (XJ) province is a resource-poor region in western China, and we believe that these two regions are representative to some extent. None of the existing clinical observational and cohort studies contain data from China; therefore, the principal aim of this study was to investigate routine procedures for intubation in critical patients (including patient evaluation, drug prescription, preparation of difficult airway devices, and personal protective equipment (PPE)) used because of the coronavirus disease (COVID-19) pandemic and basic airway management training for ICU physicians in eastern and western China.

In addition, EI poses a very high risk of severe acute respiratory syndrome coronavirus 2 transmissions, a causative agent for COVID-19, for healthcare workers, such as critical care physicians. This virus spreads through droplets, aerosols, and contact with the mucosa; therefore, appropriate PPE and effective procedures during intubation may reduce the risk of nosocomial infection [5, 6]. We aimed to investigate the impact of the COVID-19 pandemic on the use of PPE and awareness of the need for PPE during EI among ICU physicians.

## 2. Methods

### 2.1. Survey

This survey was designed based on the existing literature and COVID-19 airway management guidelines and modified from a small-scale survey in ICUs of SZ, which was conducted in 2020 by one anesthesia and three intensivists. The study was reviewed and approved by the Ethics Committee of the Peking University Shenzhen Hospital, Approval No: IRB of Peking University Shenzhen Hospital [2021] 057th. These questionnaires were distributed to the adult ICUs of 107 (including 37 in SZ and 70 in XJ) in general, secondary, and tertiary public hospitals through the WeChat app. It consisted of 23 questions (including 4 multiple choice questions) with four sections: 3 regarding information about the respondents, 3 about airway training, 15 about the procedure of induction and tracheal intubation, and 2 about PPE use. We defined the associate chief physicians and chief physicians as the senior doctors.

### 2.2. Survey Population

Participants completed all questionnaires online from March 23, 2021, to May 20, 2021. To avoid duplication, only one entry was allowed for each WeChat account. The survey included all intensive care senior and junior physicians from the selected hospitals. Participation was voluntary and anonymous, and signed informed consent forms were obtained from all participants. Those refusing to participate in the surveys and requiring assistance from other departments for endotracheal intubation were excluded from the analysis.

### 2.3. Statistical Analysis

The responses to the returned questionnaires were entered into a Microsoft Excel file. Data analysis was performed using the statistical software SPSS 26.0. The differences in responses between participants from SZ and XJ were compared using the *χ*^2^ test or if very few physicians selected specific responses, the Fisher exact test. All comparisons were performed at a 5% significance level.

## 3. Results

A total of 72 ICUs from the 107 general public hospitals invited to participate returned the questionnaire; of these, 11 were university hospitals (five from SZ and six from XJ). Excluding the 129 respondents with non-ICU specialties and those who did not perform tracheal intubation independently, 301 valid questionnaires were received from 32 hospitals in SZ (*n* = 198, 66%) and 40 hospitals in XJ (*n* = 103, 34%). Compared to those from XJ, SZ respondents did not significantly differ in terms of qualification level and ICU work experience (Table 1).

### 3.1. Training

A total of 150 (50%) respondents stated that they had completed the formal airway course and a course on the emergency front of the neck airway (FONA), and only 112 (37%) participants had been trained in RSI. There was no difference in the proportion of those trained between SZ and XJ (Table 1).

### 3.2. Assessment

In total, 217 (72%) participants stated that they routinely performed difficult airway assessments, which was more common among respondents in SZ than in XJ (77% vs. 63%; *p* = 0.012). The risk of aspiration was assessed by 194 (64%) respondents before tracheal intubation, and SZ versus XJ was not significantly different (Table 1).

### 3.3. Preoxygenation and Position

The majority of respondents preferred manual ventilation (*n* = 156, 68%) and noninvasive positive pressure ventilation (NIPPV) (*n* = 103, 34%) as preoxygenation methods. High-flow nasal cannula oxygen therapy (HFNO) was used seldomly (Table 2).

Table 2 also shows that the supine position was preferred by all respondents we surveyed. Only 63 (21%) participants answered that they used a ramped position for patients with the risk of reduced functional residual capacity (FRC), such as obese or late-term pregnancy.

### 3.4. Drugs Used in Intubation

**Figure 1:**
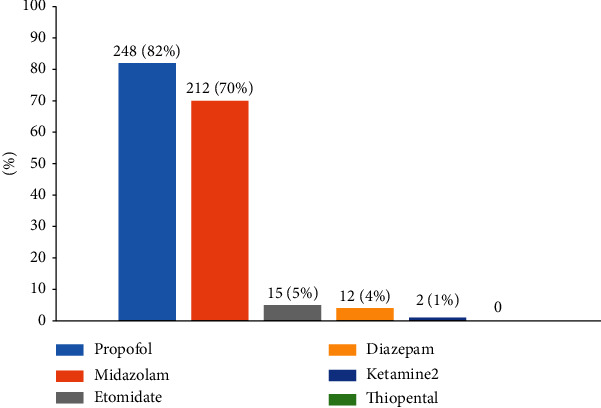
What are your preferred sedative medications for induction intubation? Multiple choices.

Propofol (*n* = 2448, 82%) and midazolam (*n* = 212, 70%) were the most commonly used agents for induction, and etomidate and ketamine were rarely administered (Figure 1) by ICU physicians.

**Figure 2:**
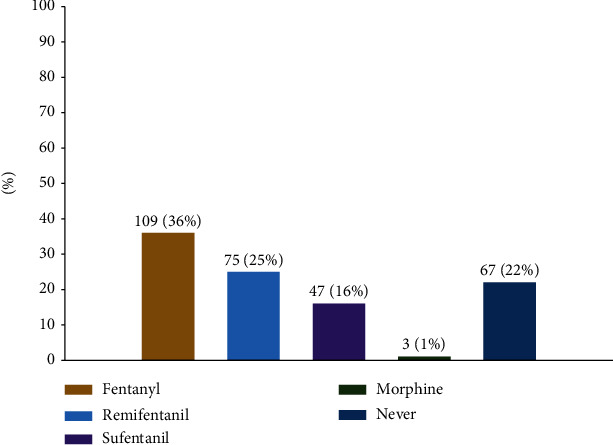
Which opioid agents are your first choice as the induction?

Figure 2 illustrates that most respondents (*n* = 234, 78%) chose to administer opioids. The preferred opioids for induction were fentanyl (*n* = 109, 36%) and remifentanil (*n* = 75, 25%).

**Figure 3:**
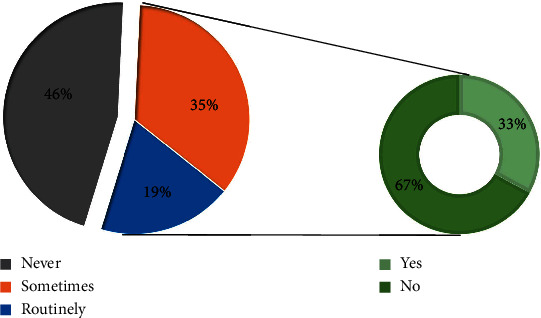
(a) Do you use neuromuscular blocker agents (NMBAs) for tracheal intubation? (b) Do you prepare reversal agents for NMBAs?

Most ICU physicians (*n* = 245, 81%) replied that they did not routinely administer NMBAs during induction. The primary reasons were “not necessary to use NMBAs” (*n* = 97, 42%) and “worried about fatal hypoxemia” (*n* = 52, 23%). Reversal NMBA (e.g., sugammadex) agents were often prepared during induction by 53 (33%) of 161 respondents who administered NMBAs (Figure 3).

### 3.5. Cricoid Pressure

**Figure 4:**
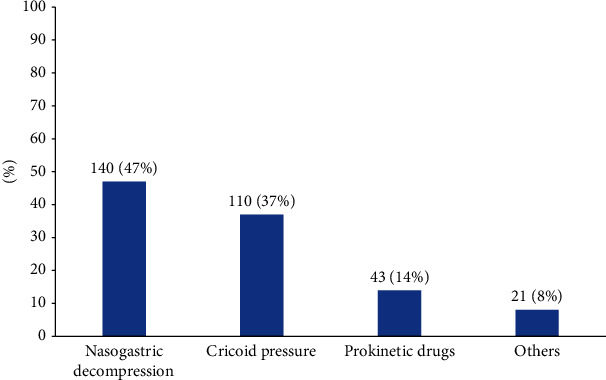
Which strategies are your choice to avoid regurgitation/aspiration? Multiple choices.

Approximately half of the respondents (*n* = 140, 47%) commonly used nasogastric decompression to prevent regurgitation or aspiration. Cricoid pressure was used by 37% (*n* = 110), and the usage rate of prokinetic drugs was only 14% (*n* = 43) (Figure 4).

### 3.6. Difficult Airway Management

Table 1 shows that a flexible endoscope was the most popular choice (76%) for preparing a difficult airway, and responses were similar between participants from SZ and XJ (84% vs. 59%; *p* ≤ 0.001). However, XJ respondents preferred to select a laryngeal mask (51% vs. 28%; *p* ≤ 0.001), while SZs were more likely to receive emergency FONA equipment (55% vs. 34%; *p* = 0.001).

### 3.7. Laryngoscope

The majority of intensivists surveyed (*n* = 286, 95%) usually employed orotracheal intubation, and 163 (82%) respondents from SZ used video laryngoscopy as the first-line choice during intubation, which was significantly different (*p* ≤ 0.001) from the practice of doctors in XJ who usually used direct laryngoscopy (43%) (Table 1).

### 3.8. PPE

**Figure 5:**
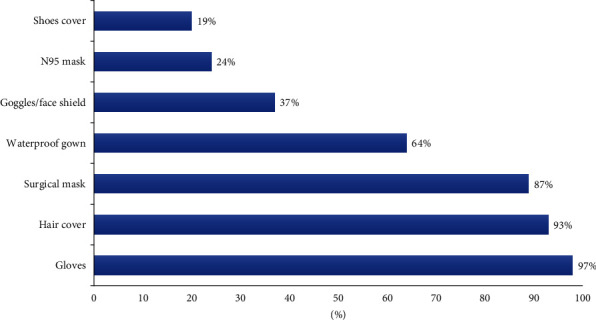
Which personal protective equipment (PPE) do you often use during intubation for the past year? Multiple choices.

Most respondents (*n* = 231, 77%) from SZ and XJ admitted that the COVID-19 pandemic had improved their awareness regarding the need for PPE during endotracheal intubation. This was reflected in the extensive application of waterproof gowns (64%), goggles/face shields (37%), and N95 masks (24%), in addition to conventional PPE (Figure 5).

## 4. Discussion

This is the first survey on the practice of EI by intensive physicians in China, and residency training is in its early stages. Our survey demonstrated that equipment availability for difficult airway management is associated with the medical resources in both regions, while there was no significant difference in the level of training between RSI and emergent FONA. Other major outcomes in our investigation include less choice of NMABs and inadequate preparation of difficult airway devices during EI. Additionally, our investigation revealed that the COVID-19 pandemic significantly improved the operator awareness of PPE requirements during EI. In addition to surgical masks, gloves, and hair covers, they also used waterproof gowns, goggles/face shield, and N95 masks, which are widely recommended during the COVID-19 pandemic [7, 8].

EI is a high-risk procedure for critically ill patients, and relevant complications in the intubation procedure decrease with improved first-attempt success, shorter intubation duration, lower risk of aspiration, and well-managed difficult airway. Meanwhile, a high first-attempt success rate limits exposure to the transmission of bacteria and viruses during intubation.

RSI is designed to facilitate rapid tracheal intubation and control the risk of aspiration. Regarding the actual reduction of adverse events during peri-intubation and the improvement of first-pass success, however, there is an ongoing debate [9, 10]. Current evidence showed significant variation and modifications in the RSI technique, including optimal position and respiratory support methods for preoxygenation, new inducing agents, and muscle relaxants [11]. Modified RSI is far from classical procedures, but the principle is widely accepted and was the recommended method for EI during the COVID-19 pandemic [12]. Our survey demonstrated that 50% (*n* = 150) of ICU physicians had formal airway training and 37% had RSI training, which is lower than UK anesthesiologists [13] and similar to Australia and New Zealand [14].

Airway assessment, including the assessment of the risk of difficult intubation and aspiration, is essential before induction. Our survey revealed that 64% of doctors routinely assessed the aspiration risk, and 72% assessed the difficult airways. Although anatomical evaluation of the airway in critically ill patients is often difficult because of the scarce and unstable functional reserve, prior assessment of the airway (absent in 22.8%) is proposed even in emergencies [15].

Hypoxemia is a common and severe complication during intubation in critically ill patients and may increase the risk of cardiac arrest and death. Although the classical RSI suggests that positive pressure ventilation should be avoided during induction, increasing evidence has proved that NIPPV was more effective than usual preoxygenation in improving oxygen saturation and reducing the incidence of severe hypoxemia [16, 17]. A multicenter, randomized, open-label trial stated that noninvasive ventilation was equivalent to HFNO use for preoxygenation in patients with mild respiratory failure but maybe better than HFNO use in patients with a PaO2/FiO2 ratio < 200 mmHg [18]. Therefore, HFNO and NIPPV are recommended for airway management in ICUs for patients with acute hypoxemia, and NIPPV should be the preferred option for patients with severe hypoxemia [15, 19, 20]. In light of this, it is reasonable that our responders' first-line rescue strategies for preoxygenation before intubation were NIPPV (34%) and manual ventilation (56%).

The ramped position has been shown to improve blood saturation in both obese and nonobese patients with impaired FRC [21, 22] and reduce the risk of aspiration [23]. Although there is some contention regarding whether the ramped position is associated with increased intubation difficulty compared to the sniffing position [24, 25], current guidelines remain to recommend a head-up position for preoxygenation, especially in patients at high risk of aspiration or desaturation [15, 19, 20]. Our survey found that only a minority of ICU physicians adhered to the recommendation for the use of the head-up position, which may be attributable to the relatively low proportion of overweight patients in China.

Aspiration is another major complication that should be avoided. Our responders preferred nasogastric decompression to reduce aspiration (47%) rather than cricoid pressure (37%), which is parallel to the results of the INTUBE study [2, 13]. In the classical RSI, the Sellick maneuver (cricoid pressure) is a basic method used to prevent aspiration. However, the largest randomized trial did not find any superiority in performing cricoid force in patients undergoing anesthesia with RSI [26], and this manoeuver can deteriorate the visual field for laryngoscopy and require a specific training to accomplish [27]. Furthermore, during induction for patients with COVID-19, the risk-benefit of applying cricoid pressure should be carefully considered as it can stimulate coughing; therefore, it is no longer mandatory as per the guidelines for difficult airway management and patients with COVID-19 [15, 28, 29].

Nasogastric decompression seems to be a simple and affordable intervention to evacuate gastric contents to reduce the risk of regurgitation/aspiration; but there are only limited data about the requirement for a gastric tube before anesthesia induction and the appropriate gastric tube management in RSI [13].

The peri-intubation cardiovascular collapse was the most common adverse event in the INTUBE study. Although ketamine was the first-line recommendation for hemodynamic instability patients during intubation due to its superior hemodynamic effect [30], a recent prospective randomized single-center study reported that for emergency EI, etomidate was comparable to ketamine in the 28 mortality rate. Nevertheless, propofol and midazolam were the most frequently prescribed as induction agents [31, 32], and our investigation revealed a similar outcome, with lower rates of ketamine and etomidate use. This may be attributable to the fact that ketamine is currently unavailable in China, as well as concerns regarding etomidate's adrenal suppression in sepsis patients.

Opioids are not the classical induction agents during RSI, but they could reduce the cardiovascular response to laryngoscopy, the dose of other induction agents, and intracranial pressure fluctuations. 74% of our respondents indicated that they administered opioids; this result is slightly more than the choice of ICU physicians in other countries (51%) [2]. Opioids have also been recommended for awake EI for difficult airways [33] owing to high levels of patient satisfaction and low risk of oversedation and airway obstruction [34].

In comparison to other nations' survey data, we discovered that just 19% of intensivists routinely prescribed NMBAs in RSI, which is the key difference. The use of NMBAs is recommended in airway management guidelines developed by anesthesiologists as it facilitates intubation in ICUs to increase first-attempt intubation success rates [5, 28, 29, 35]. Although two systematic reviews found that the use of NMBAs can improve the conditions for EI and reduce number of difficult airway and intubation complications [36, 37], there are misgivings regarding the use of NMBAs in critically ill patients. First, current evidence from emergency departments and ICUs is still sparse. Second, given the physiologically difficult airway of critically ill patients, the clinicians are afraid of a “cannot intubate, cannot ventilate” scenario and secondary fatal desaturation. Therefore, patients who are at risk for refractory hypoxemia, difficult intubation, and problematic ventilation (facial mask/supraglottic airway SGA) should undergo awake intubation rather than NMBAs [38]. However, we must highlight that awake intubation in individuals with hypoxemia requires the cooperation of the patient, or else the patient may get agitated [30]; thus, a highly trained and skilled team is required. More high-quality RCT studies are needed to evaluate the safety of NMBAs in the airway management for critically ill patients. Meanwhile, due to the increased risk of coronavirus for healthcare workers during intubation, clinicians were suggested to apply the NMBAs and avoid performing awake intubation in patients with COVID-19 [28, 29, 39].

If there is concern about being unable to ventilate during the apneic time, the availability of sugammadex, a drug that rapidly reverses the effects of steroidal NMBAs [40], may render rocuronium a more attractive option for optimizing intubating conditions. However, the high cost limits its application.

Surveys on the use of NMBAs in ICUs have shown significant variability across countries [2, 14, 31, 41]. In our survey, a large proportion of participants replied that they did not adopt NMBAs because they did not feel the necessity (43%). We speculate that the large use (75%) of video laryngoscopy (VL) may contribute to intensivists' confidence in the first-attempt success of intubation, which is significantly higher than the data from other countries [2]. Although a large multicenter randomized controlled trial performed in ICU reported that a combo VL compared to DL did not increase the first-attempt success rate [42], which was considered to be due to inadequate training of operators in further analysis, the recent systematic review elucidated that VLs were likely to reduce the rate of failed intubation and result in a higher rate of successful intubation on the first attempt with improved glottic views [43]. Furthermore, VL provides conditions for reducing the spread of aerosols during intubation. Therefore, the current guidelines highlight that a video laryngoscope should be available [5, 33] and considered as a first-line option for all intubations of critically ill patients undergoing COVID-19 [6, 8, 12]. The distinction in video laryngoscope use between respondents from SZ (82%) and XJ (50%), as revealed in our study, may be explained by the availability of resources.

The incidence of difficult intubation was about 5%, and the failed EI after two attempts was 4.6% in critically ill patients [2]. The latest reviews and guidelines emphasize the importance of having SGAs and FONA kits available for all patients, especially when a difficult airway is suspected or intubation has failed [38, 44]. Following successful SGA insertion and ventilation, fiberoptic-guided intubation via second-generation SGA is recommended [15]. Our data showed that just 36% of respondents had access to SGA, while 48% had access to FONA kits. These devices are also not common in all ICUs around the world [31].

### 4.1. Limitations

Our study had some limitations. First, the surveyed population only included two regions in China, which limited representativeness. Second, the proportion of hospitals surveyed in XJ was not as high as in SZ, leading to a bias. Third, our questionnaire was voluntarily completed by doctors in the ICU of each hospital, and random sampling was not used, which may have biased the results. Fourth, we did not inquire about the checklist preparation before intubation, which is considered one of the important steps to improve the success rate of intubation. Fifth, this study did not collect more information on difficult airway management, including serial preparation protocols, apneic oxygenation techniques, and awake EI.

#### 4.1.1. Further Directions

The focus of future research should be the collection of larger adverse event data and the management of difficult airways in critically ill patients in China. The protective effect of nasogastric decompression in patients with high-risk aspiration merits further evaluation, and optimal induction drugs should be confirmed by high-quality, well-powered RCTs, especially in septic patients undergoing EI. Further clinical practice and studies should focus on specific procedures and PPEs to protect healthcare workers performing intubation on critically ill patients, even in the absence of a pandemic.

## 5. Conclusions

In this first survey study on intubation practice in Chinese intensivists from two regions, the findings revealed a considerable scope for improvements in EI training in Chinese ICUs. Despite some differences in medical equipment resources across regions, there was little variation in the training and practice of EI between SZ and XJ. One of the obvious results was the low application of NMBAs; therefore, it is necessary to establish a quality monitoring system and guidelines for EI in critically ill patients to strengthen unified training in China.

## Figures and Tables

**Table 1:** Type of hospital, qualification, work experience, training, assessment, intubation method, and device available for difficult airway.

	Total (%)	Shenzhen (%)	Xinjiang (%)	*p* value
ICU respondents	301	198 (66)	103 (34)	
Type of hospital	72	32	40	
University hospital	11	6	5	
District hospital	61	26	35	0.552
Level of qualification				
Junior	214 (71)	139 (70)	75 (73)	
Senior	87 (29)	59 (30)	28 (27)	0.635
Years of work in ICU				
1-6 years	174 (58)	114 (58)	58 (56)	
≥7 years	127 (42)	84 (42)	45 (44)	0.833
Formal airway management training: yes	150 (50)	99 (50)	51 (50)	0.936
Training of RSI: yes	112 (37)	81 (40)	31 (30)	0.066
Training of emergency FONA: yes	150 (50)	94 (47)	56 (54)	0.256
Difficult airway assessment				
Routinely	218 (72)	153 (77)	65 (63)	0.012
Sometimes	75 (25)	39 (20)	36 (35)	0.005
Never	8 (3)	6 (3)	2 (2)	0.720
Aspiration risk assessment				
Routinely	194 (64)	132 (67)	62 (60)	0.266
Sometimes	74 (25)	41 (20)	33 (32)	0.030
Never	33 (11)	25 (13)	8 (8)	0.245
Tracheal intubation approach				
Orotracheal intubation	286 (95)	186 (94)	100 (97)	
Nasotracheal intubation	16 (5)	12 (6)	3 (3)	0.234
The routine choice for laryngoscope glottic view				
Video laryngoscope	225 (75)	163 (82)	52 (50)	≤0.001
Direct laryngoscope	59 (20)	25 (13)	44 (43)	≤0.001
Flexible scope	17 (5)	10 (5)	7 (7)	0.719
An available laryngeal mask: yes	108 (36)	55 (28)	53 (51)	≤0.001
An available flexible endoscope: yes	228 (76)	167 (84)	61 (59)	≤0.001
An available device for FONA: yes	144 (48)	109 (55)	35 (34)	0.001
COVID-19 improve the usage of PPE?				
Significant	231 (77)	147 (74)	84 (81)	0.154
A little	63 (31)	47 (24)	16 (16)	0.097
Not at all	7 (2)	4 (2)	3 (3)	0.694

Abbreviations: ICU: intensive care unit; RSI: rapid sequence intubation; FONA: front of neck airway; PPE: personal protective equipment.

**Table 2:** Position and method for preoxygenation.

Question	Number	Percentage
Position for preoxygenation		
Routine supine position	301	100%
Routine ramped position	0	0
Ramped position for patients with impaired FRC, e.g., obese or late-term pregnancy	63	21%
How to preoxygenate		
Nasal tube	2	1%
Face mask	19	6%
Manual ventilation	169	56%
NIPPV	103	34%
HFNO	8	3%

Abbreviations: FRC: functional residual capacity; NIPPV: noninvasive positive pressure ventilation; HFNO: high-flow nasal cannula oxygen therapy.

## Data Availability

The data used to support the findings of this study could be provided if required by editors and reviewers.

## References

[1] Cook T. M. (2018). Strategies for the prevention of airway complications – a narrative review. *Anaesthesia*.

[2] Russotto V., Myatra S. N., Laffey J. G. (2021). Intubation practices and adverse peri-intubation events in critically ill patients from 29 countries. *JAMA*.

[3] Mosier J. M., Sakles J. C., Law J. A., Brown C. A., Brindley P. G. (2020). Tracheal intubation in the critically Ill. Where we came from and where we should go. *American Journal of Respiratory and Critical Care Medicine*.

[4] Karamchandani K., Wheelwright J., Yang A. L., Westphal N. D., Khanna A. K., Myatra S. N. (2021). Emergency airway management outside the operating room: current evidence and management strategies. *Anesthesia and Analgesia*.

[5] Contini C., Caselli E., Martini F. (2020). COVID-19 is a multifaceted challenging pandemic which needs urgent public health interventions. *Microorganisms*.

[6] Lockhart S. L., Duggan L. V., Wax R. S., Saad S., Grocott H. P. (2020). Personal protective equipment (PPE) for both anesthesiologists and other airway managers: principles and practice during the COVID-19 pandemic. *Canadian Journal of Anesthesia*.

[7] Wax R. S., Christian M. D. (2020). Practical recommendations for critical care and anesthesiology teams caring for novel coronavirus (2019-nCoV) patients. *Canadian Journal of Anesthesia*.

[8] Alhazzani W., Møller M. H., Arabi Y. M. (2020). Surviving sepsis campaign: guidelines on the management of critically ill adults with coronavirus disease 2019 (COVID-19). *Critical Care Medicine*.

[9] Okubo M., Gibo K., Hagiwara Y., Nakayama Y., Hasegawa K. (2017). The effectiveness of rapid sequence intubation (RSI) versus non-RSI in emergency department: an analysis of multicenter prospective observational study. *International Journal of Emergency Medicine*.

[10] Wallace C., McGuire B. (2014). Rapid sequence induction: its place in modern anaesthesia. *Continuing Education in Anaesthesia Critical Care & Pain*.

[11] Lyon R. M., Perkins Z. B., Chatterjee D., Lockey D. J., Russell M. Q., on behalf of Kent, Surrey & Sussex Air Ambulance Trust (2015). Significant modification of traditional rapid sequence induction improves safety and effectiveness of pre-hospital trauma anaesthesia. *Critical Care*.

[12] Ong S., Lim W. Y., Ong J., Kam P. (2020). Anesthesia guidelines for COVID-19 patients: a narrative review and appraisal. *Korean Journal of Anesthesiology*.

[13] Zdravkovic M., Berger-Estilita J., Sorbello M., Hagberg C. A. (2020). An international survey about rapid sequence intubation of 10,003 anaesthetists and 16 airway experts. *Anaesthesia*.

[14] Toolis M., Tiruvoipati R., Botha J., Green C., Subramaniam A. (2019). A practice survey of airway management in Australian and New Zealand intensive care units. *Critical Care and Resuscitation*.

[15] Natt B., Mosier J. (2021). Airway management in the critically ill patient. *Current Anesthesiology Reports*.

[16] Casey J. D., Janz D. R., Russell D. W. (2019). Bag-mask ventilation during tracheal intubation of critically ill adults. *The New England Journal of Medicine*.

[17] Baillard C., Prat G., Jung B. (2018). Effect of preoxygenation using non-invasive ventilation before intubation on subsequent organ failures in hypoxaemic patients: a randomised clinical trial. *British Journal of Anaesthesia*.

[18] Frat J. P., Ricard J. D., Quenot J. P. (2019). Non-invasive ventilation versus high-flow nasal cannula oxygen therapy with apnoeic oxygenation for preoxygenation before intubation of patients with acute hypoxaemic respiratory failure: a randomised, multicentre, open-label trial. *The Lancet Respiratory Medicine*.

[19] Lapinsky S. E. (2015). Endotracheal intubation in the ICU. *Critical Care*.

[20] Kornas R. L., Owyang C. G., Sakles J. C., Foley L. J., Mosier J. M., on behalf of the Society for Airway Management’s Special Projects Committee (2021). Evaluation and management of the physiologically difficult airway: consensus recommendations from society for airway management. *Anesthesia and Analgesia*.

[21] Petrini F., Di Giacinto I., Cataldo R. (2016). Perioperative and periprocedural airway management and respiratory safety for the obese patient: 2016 SIAARTI consensus. *Minerva Anestesiologica*.

[22] Ramkumar V., Umesh G., Philip F. A. (2011). Preoxygenation with 20º head-up tilt provides longer duration of non-hypoxic apnea than conventional preoxygenation in non-obese healthy adults. *Journal of Anesthesia*.

[23] Khandelwal N., Khorsand S., Mitchell S. H., Joffe A. M. (2016). Head-elevated patient positioning decreases complications of emergent tracheal intubation in the ward and intensive care unit. *Anesthesia and Analgesia*.

[24] Turner J. S., Ellender T. J., Okonkwo E. R. (2017). Feasibility of upright patient positioning and intubation success rates at two academic EDs. *The American Journal of Emergency Medicine*.

[25] Semler M. W., Janz D. R., Russell D. W. (2017). A multicenter, randomized trial of ramped position vs sniffing position during endotracheal intubation of critically ill adults. *Chest*.

[26] Birenbaum A., Hajage D., Roche S. (2019). Effect of cricoid pressure compared with a sham procedure in the rapid sequence induction of anesthesia: the IRIS randomized clinical trial. *JAMA Surgery*.

[27] White L., Thang C., Hodsdon A., Melhuish T., Vlok R. (2020). Cricoid pressure during intubation: a systematic review and meta-analysis of randomised controlled trials. *Heart & Lung*.

[28] Sorbello M., El‐Boghdadly K., Di Giacinto I. (2020). The Italian coronavirus disease 2019 outbreak: recommendations from clinical practice. *Anaesthesia*.

[29] Triplett K. E., Collett L. W. (2021). Consensus statement: Safe Airway Society principles of airway management and tracheal intubation specific to the COVID-19 adult patient group. *The Medical Journal of Australia*.

[30] Higgs A., McGrath B. A., Goddard C. (2018). Guidelines for the management of tracheal intubation in critically ill adults. *British Journal of Anaesthesia*.

[31] Mendes P. V., Besen B., Lacerda F. H., Ramos J., Taniguchi L. U. (2020). Neuromuscular blockade and airway management during endotracheal intubation in Brazilian intensive care units: a national survey. *Revista Brasileira de Terapia Intensiva*.

[32] Matchett G., Gasanova I., Riccio C. A. (2022). Etomidate versus ketamine for emergency endotracheal intubation: a randomized clinical trial. *Intensive Care Medicine*.

[33] Frerk C., Mitchell V. S., McNarry A. F. (2015). Difficult Airway Society 2015 guidelines for management of unanticipated difficult intubation in adults. *British Journal of Anaesthesia*.

[34] Johnston K. D., Rai M. R. (2013). Conscious sedation for awake fibreoptic intubation: a review of the literature. *Canadian Journal of Anesthesia*.

[35] Cook T. M., El-Boghdadly K., McGuire B., McNarry A. F., Patel A., Higgs A. (2020). Consensus guidelines for managing the airway in patients with COVID-19. *Anaesthesia*.

[36] Lundstrøm L. H., Duez C., Nørskov A. K. (2018). Effects of avoidance or use of neuromuscular blocking agents on outcomes in tracheal intubation: a Cochrane systematic review. *British Journal of Anaesthesia*.

[37] Lundstrøm L. H., Duez C. H. V., Nørskov A. K. (2017). Avoidance versus use of neuromuscular blocking agents for improving conditions during tracheal intubation or direct laryngoscopy in adults and adolescents. *Cochrane Database of Systematic Reviews*.

[38] Apfelbaum J. L., Hagberg C. A., Connis R. T. (2022). 2022 American Society of Anesthesiologists practice guidelines for management of the difficult airway. *Anesthesiology*.

[39] Zuo M., Huang Y., Ma W. (2020). Expert recommendations for tracheal intubation in critically ill patients with noval coronavirus disease 2019. *Chinese Medical Sciences Journal*.

[40] Keating G. M. (2016). Sugammadex: a review of neuromuscular blockade reversal. *Drugs*.

[41] Simpson G. D., Ross M. J., McKeown D. W., Ray D. C. (2012). Tracheal intubation in the critically ill: a multi-centre national study of practice and complications. *British Journal of Anaesthesia*.

[42] Lascarrou J. B., Boisrame-Helms J., Bailly A. (2017). Video laryngoscopy vs direct laryngoscopy on successful first-pass orotracheal intubation among ICU patients: a randomized clinical trial. *JAMA*.

[43] Hansel J., Rogers A. M., Lewis S. R., Cook T. M., Smith A. F. (2022). Videolaryngoscopy versus direct laryngoscopy for adults undergoing tracheal intubation. *Cochrane Database of Systematic Reviews*.

[44] Heidegger T. (2021). Management of the difficult airway. *The New England Journal of Medicine*.

